# Associations between cognitive impairment and motor dysfunction in Parkinson's disease

**DOI:** 10.1002/brb3.719

**Published:** 2017-05-12

**Authors:** Yi‐Xuan Wang, Jue Zhao, Da‐Ke Li, Fang Peng, Ying Wang, Ke Yang, Zhen‐Yang Liu, Feng‐Tao Liu, Jian‐Jun Wu, Jian Wang

**Affiliations:** ^1^ Department & Institute of NeurologyHuashan Hospital & National Clinical Research Center for Aging and MedicineFudan UniversityShanghaiChina

**Keywords:** association, cognitive impairment, motor dysfunction, Parkinson's disease

## Abstract

**Introduction:**

Numerous studies have been carried out to explore the potential association between neurologic deficits and variable clinical manifestations of Parkinson's disease (PD). The aim of our study was to investigate the association between cognitive performance and motor dysfunction in Chinese patients with PD.

**Methods:**

Data from 96 patients with PD were obtained from the Parkinson's disease patient cohort database of Huashan Hospital. All participants underwent a comprehensive neuropsychological evaluation to assess cognitive status, that included scoring on the Mini‐mental state examination (MMSE), followed by more detailed cognitive assessment on five main cognitive domains (verbal memory, nonverbal memory, visuospatial function, language and attention/executive function). Correlations between cognitive and motor scores were investigated after controlling for age, disease duration, education, and gender.

**Results:**

We report a significant correlation between subdomains of cognitive impairment and motor dysfunction using analyses of the multiple linear regression. Notably, executive function and attention was significantly associated with bradykinesia and rigidity, while visuospatial function was associated with bradykinesia and tremor.

**Conclusions:**

The association between motor dysfunction and cognitive decline in PD might highlight deficits represented by a shared neurochemical pathway.

## 1 Introduction

It is well recognized that Parkinson's disease (PD) is a multifaceted neurodegenerative disorder characterized by both motor (bradykinesia, resting tremor, rigidity, and postural instability) and non‐motor symptoms (REM behavior disorder [RBD], hyposmia, constipation, depression and, cognitive impairment) (Chaudhuri, Healy, & Schapira, 2006). Even in early stages of PD, cognition is commonly impacted on a range of subdomains and with substantial heterogeneity. Among them, problems with executive function, attention/working memory, and visuospatial function are consistently revealed by a battery of neuropsychological examinations in early PD (Aarsland, Bronnick, Larsen, Tysnes, & Alves, 2009; Elgh et al., 2009).

The relationships between different PD motor phenotypes and specific cognitive dysfunction have been investigated in de novo and medicated PD patients. In several early investigations, postural instability and gait difficulties (PIGD) subtype was prone to a faster rate of cognitive decline (Williams‐Gray, Foltynie, Brayne, Robbins, & Barker, 2007), while tremor‐dominant subtype was less likely to develop cognitive impairment (Alves, Larsen, Emre, Wentzel‐Larsen, & Aarsland, 2006; Burn et al., 2006). The connections between motor functions and cognitive domains have been investigated in newly diagnosed, drug naive PD patients. These authors found a shared system for bradykinesia and inflexible thinking, no association was found between cognitive performance and tremor (Domellof, Elgh, & Forsgren, 2011; Poletti et al., 2012). However, cognitive deficits may be subtle in newly diagnosed, drug naive PD patients and no consensus has been reached with respect to the structured cognitive profiles associated with the motor manifestations in PD. In addition, the effect culture may have on cognitive assessment, in particular Chinese culture, has yet to be examined. Herein, the objective of this study is to investigate the association between motor symptom subtypes and profiles of cognitive function in Chinese nondemented PD patients.

## 2 Methods

### 2.1 Subjects

All participants enrolled in the study were patients under care at the Movement Disorders Clinic of Huashan Hospital affiliated to Fudan University. Their data were collected from a Parkinson's disease database produced within the clinic. All participants fulfilled the criteria for idiopathic PD (iPD) according to UK Parkinson's Disease Society Brain Bank criteria (Hughes, Daniel, Kilford, & Lees, 1992). Diagnoses were confirmed after being followed for 2–6 years. Atypical Parkinsonism, such as multiple system atrophy, progressive supranuclear palsy or corticobasal degeneration, were excluded.

For those late‐onset PD patients with onset being above 40 years and H&Y stage being I‐IV, there were 221 consecutive patients with idiopathic Parkinson's disease who agreed to participate in this study during the period of July 2010 to March 2014, and further assessments were performed after informed consents. Diagnosis for five patients was changed during follow‐up. Those patients (*n* = 31) who underwent UPDRS‐III rating on anti‐PD medications were not included in this study, nor were those patients with total UPDRS‐III score less than 7 (*n* = 5) during off‐medications. An additional 38 patients declined or were not able to finish the neuropsychological assessments, therefore, 142 PD patients in total finished neuropsychological evaluation. Of those PD patients, a subset on anticholinergic medication or with dementia defined as MMSE score being below 24 were also excluded from this study. Thus, 96 PD patients were included in the analyses.

All participants provided written informed consent which was in accordance with the Declaration of Helsinki and approved by the Human Studies Institutional Review Board at Huashan Hospital affiliated to Fudan University.

### 2.2 Assessment

All participants underwent a comprehensive clinical assessment including motor and cognitive testing. Information on demographics, antiparkinsonian medication and medical history was obtained. To standardize data on medication, we converted dosages of PD medications to total daily levodopa equivalent doses. The motor part of the UPDRS (Part III) was used to assess the severity of disease by two independent trained interviewers during an “OFF” medication state, which was defined as being off antiparkinsonism medications for at least 12 hr. Meanwhile, a videotaping of the patient undergoing UPDRS–motor score evaluation was recorded for a possible later review. The motor subscores were calculated from the UPDRS‐III: the sum of UPDRS items 20 and 21 for the tremor score, item 22 for rigidity, the sum of items 23, 24, 25, and 26 for the bradykinesia score, the sum of items 27, 28, 29, and 30 for the posture and gait score (arising from chair, posture, gait, and postural stability), and the sum of items 18 and 19 for the bulbar score (speech and facial expression).

After being evaluated on motor dysfunction in the morning, patients were then required to take their regular antiparkinsonian medications, and cognitive evaluation was conducted during the “ON” state. Global cognition was assessed using the Mini Mental State Examination (MMSE) (Katzman et al., 1988) and depression was rated using the Geriatric Depression Rating Scale (GDS) (Yesavage et al., 1982) and Beck Depression Inventory (BDI) (Beck, Ward, Mendelson, Mock, & Erbaugh, 1961). Five specific cognitive domains were assessed by a comprehensive neuropsychological battery, with attention being tested by the Symbol Digit Modality Test (SDMT) (Sheridan et al., 2006) and Trail Making Test A (TMT‐A) (Zhao et al., 2013); executive function by the Stroop Color‐Word Test (CWT) (Steinberg, Bieliauskas, Smith, & Ivnik, 2005) and the Trail Making Test B (TMT‐B) (Zhao et al., 2013); language by the Boston Naming Test (BNT) (Lucas et al., 2005) and Verbal Fluency Test (VFT, animals, cities, alternatives) (Lucas et al., 2005); memory by the Auditory Verbal Learning Test (AVLT) (Guo, Zhao, Chen, Ding, & Hong, 2009) and delayed recall of the Rey‐Osterrieth Complex Figure Test (CFT‐recall)(Caffarra, Vezzadini, Dieci, Zonato, & Venneri, 2002); and visuospatial function by the Clock Drawing Test (CDT) (Wang, Shi, Zhao, Hong, & Guo, 2014) and copy task of Rey‐Osterrieth Complex Figure test(CFT‐copy) (Caffarra et al., 2002).

### 2.3 Statistical analysis

For all statistical analyses, we used Statistical Package SPSS version 22.0(SPSS, RRID:SCR_002865). Since most variables were not normally distributed, the nonparametric Spearman's rho was used to explore correlations between motor and cognitive variables. Independent demographic variables found correlated with psychological variables were used as covariates and forced into a stepwise multivariable liner regression analysis to determine if the relationships found between the motor and neuropsychological variables were unique. Raw scores from each of the neuropsychological tests were used as the dominant variable in separate multiple linear regression models. Multicollinearity diagnostics were performed and residual distribution was checked. A *p*‐value less than .05 was taken to indicate a statistically significant relationship between the corresponding pair of variables interrogated.

## 3 Results

The demographic and clinical characteristics of 96 participants are shown in Table 1. Neuropsychological tests performed on the participants took, on average, 1 hr. The majority of participants completed all assessments, with the exceptions that four did not complete the CFT memory test and six patients quit the AVLT recognition component during assessment.

**Table 1:** Demographics characteristics

	PD subjects (*n* = 96), mean(SD)
Age of onset	57.9 (8.0)
Age of assessment, y	61.8 (7.9)
Sex(M/F)	55/41
Education, y	13.1 (3.0)
PD duration (months)	46.8 (47.2)
Hoehn & Yahr stage	1.9 (0.7)
UPDRS–III	23.9 (10.8)
Tremor	3.7 (3.5)
Rigidity	10.6 (5.0)
Bradykinesia	4.1 (3.0)
PIGD	2.0 (0.8)
Bulbar	2.0 (1.2)
MMSE	28.3 ± 1.8
Total equivalent dose	222.0 (299.7)
Levodopa treatment	155.0 (243.8)
Dopamine agonist	32.8 (59.3)
MAOB‐I	12.2 (32.2)

UPDRS, Unified Parkinson's Disease Rating Scale; PIGD, postural instability and gait disorders; MMSE, Mini‐mental state examination; MAOI‐B, monoamine oxidase inhibitor type B.

Using nonparametric correlations (Table 2), cognitive function was significantly correlated with age, gender, disease duration, and years of education. UPDRS sub‐items (bradykinesia, rigidity, and axial symptoms) were correlated with a range of neuropsychological measures, but tremor was not correlated with any cognitive measure. In the multiple linear regression analysis, with age, disease duration, sex, education and the other motor signs being controlled for in 15 models, the following cardinal signs contributed to the model (Table 3): tremor was positively correlated with delayed recall score of CFT (β = .295, *p* = .006); bradykinesia was significantly correlated with CFT copy time (β = .277, *p* = .005), delayed recall score of CFT (β = −.358, *p* = .001), SDMT (β = −.258, *p* = .008), TMT‐A (β = .442, *p* < .001), TMT‐B (β = .304, *p* = .001) and VFT animal/word interference test (β = −.460, *p* < .001); rigidity was positively correlated with VFT animal/word interference test (β = .301, *p* = .017) and CDT (β = .284, *p* = .008). In addition, negative correlations were found between PIGD subscore and CDT (β = −.330, *p* = .002) and Stroop color word interference test (β = .258, *p* = .008), respectively. No correlation was found between bulbar score and cognitive measures. AVLT and BNT did not correlate with any motor subscore.

**Table 2:** Correlations between Neuropsychological performance and Off UPDRS motor scores (spearman rho, *n* = 96)

Cognitive domain	Neuropsychological tests	Education	Sex	Age	Disease Duration	Tremor	Bradykinesia	Rigidity	PIGD	Bulbar
Visuospatial function	CFT ‐ copy time	−.150	.054	.147	.162	.193	.282^b^	.283^b^	.154	.184
	CFT ‐ copy score	.248^a^	−.006	.166	−.220^a^	.030	−.250^a^	−.085	−.204^a^	−.121
	CDT	−.033	−.206^a^	.034	−.123	.056	−.009	.171	−.199	−.128
Executive function	Stroop‐word	−.296^b^	.301^b^	.227^a^	.219^a^	.133	.235^a^	.038	.075	.228^a^
	Stroop‐color	−.152	.273^b^	.244^a^	.141	.041	.129	−.135	.058	.026
	Stroop‐color word	−.104	.167	.302^b^	.263^a^	.097	.182	−.008	.180	.094
	TMT‐B	−.189	.043	.242^a^	.319^b^	.094	.315^b^	.124	.250^a^	.085
Attention	SDMT	.139	−.075	−.182	−.147	−.046	−.313^b^	−.153	−.139	−.230^a^
	TMT‐A	−.216^a^	−.075	.369^b^	.224^a^	.143	.332^b^	.149	.177	.129
Language	BNT	.298^b^	.184	.147	−.065	−.010	−.112	−.122	−.024	.075
	VFT ‐ animal	.313^b^	.131	.030	−.065	−.106	−.129	−.019	.006	.116
	VFT ‐ city	.300^b^	.260^a^	.001	.12	−.021	−.153	−.025	−.090	.097
	VFT ‐ animal/city	.176	.026	.036	−.035	−.113	−.272^b^	−.009	−.260^a^	.041
Memory	AVLT ‐ short delayed recall	.142	−.097	−.022	−.008	.077	−.180	−.017	−.109	.027
	AVLT ‐ long delayed recall	.196	−.018	−.075	−.045	.066	−.155	.007	−.101	.096
	AVLT ‐ sum 1 to 5	.230^a^	−.058	−.048	−.063	.090	−.162	.001	−.137	.016
	AVLT ‐ recognition	.131	.203	−.103	.045	−.020	−.162	−.060	−.056	−.005
	CFT ‐ delayed recall	.267^a^	.063	.129	−.048	.161	−.237^a^	−.066	−.152	.014

CFT, Rey‐Osterrieth Complex Figure Test; CDT, Clock Drawing Test; TMT, Trail Making Test; SDMT, Symbol Digit Modality Test; BNT, Boston Naming Test; VFT, Verbal Fluency Test; AVLT, Auditory Verbal Learning Test; PIGD, postural instability and gait disorders.

a Significant correlation when confidence level is .05.

b Significant correlation when confidence level is .01.

**Table 3:** Multiple regression analysis of motor and cognitive dysfunction in 96 patients with PD

	R²	β	*p* value
Tremor			
CFT‐delayed recall	.162	.295	.006
Bradykinesia			
CFT‐copy time	.112	.277	.005
CFT‐delayed recall	.162	−.358	.001
SDMT	.142	−.258	.008
TMT‐A	.387	.442	<.001
TMT‐B	.231	.304	.001
VFT‐animal/city	.127	−.460	<.001
Rigidity			
VFT‐animal/city	.127	.301	.017
CDT	.127	.284	.008
PIGD			
Stroop‐color/word time	.184	.258	.008
CDT	.127	−.330	.002

CFT, Rey‐Osterrieth Complex Figure Test; SDMT, Symbol Digit Modality Test; TMT, Trail Making Test; VFT, Verbal Fluency Test; CDT, Clock Drawing Test.

## 4 Discussion

In this study, we investigated the associations between impairments in cognitive subdomains and motor features in Chinese patients with PD. In summary, we show that executive dysfunction, Stroop Color Word Interference Test was associated PIGD; Trail Making Test (TMT‐B) was associated with bradykinesia; and VFT‐animal/city associated with bradykinesia and rigidity. In the cognitive subdomain of attention, Symbol Digit Modality Test (SDMT) was associated with bradykinesia. In the visuospatial domain, copy time of CFT was associated with bradykinesia. In memory domain, recall score of CFT was associated with bradykinesia and tremor.

Executive function is considered critical to ones neuropsychological profile (Watson & Leverenz, 2010). In PD, the neuropathological correlates of executive dysfunction have been shown to be, in part, a result of fronto‐striatal circuit impairment. Thus, the dopaminergic deficits within the basal ganglia play a functional role in executive dysfunction. (Kehagia, Barker, & Robbins, 2010). Growing evidence points towards an association between executive dysfunction and motor manifestations, including PIGD (Amboni, Barone, & Hausdorff, 2013). However, likely due to the disparate rating tools used among investigators, not all reports are in agreement. This is especially true when considering the results from the specific subdomains of the assessment tools utilized. For example, in Uc et al. (2005), the sub‐items, instability and gait disorders, from UPDRS III were performed to evaluate PIGD. They report that poorer visuospatial abilities and executive function were both found to be associated with worse gait and postural instability (Uc et al., 2005). In contrast, in another study, axial signs (measured by UPDRS III) were associated with worse visuospatial memory and visuospatial functioning in newly diagnosed PD (Domellof et al., 2011). Vercruysse et al. (2012) found that executive function measured by the SCOPA‐COG was a significant predictor of freezing of gait (FOG), a special form of PIGD.

In our current work, we selected related sub‐items from UPDRS III to evaluate PIGD along with a comprehensive cognitive test for assessing executive dysfunction. We show that part III of the Stroop test (time of color‐word task) was positively correlated with the PIGD score. In a previous study studying PD patients with freezing of gait (FOG), there was impairments found in Stroop test part II, but no change discovered in the mean Stroop part III scores in those with FOG (Amboni, Cozzolino, Longo, Picillo, & Barone, 2008). Therefore, our findings further extend the relationship between executive dysfunction and the occurrence of PIGD in PD. The mechanism of PIGD in PD may be caused by not only dopaminergic deficits but also by other disrupted neurotransmitter system (Owen et al., 1992), since there is the commonality between the neural networks underlying these subdomains of cognitive impairments and motor features (Gratwicke, Jahanshahi, & Foltynie, 2015). Another potential underlying mechanism for FOG may be a lack of sufficient compensation of executive function in gait, since increased cognitive load was found to lead to freezing gait by utilizing dual‐task tests analogous to performing cognitive tasks while walking (Hausdorff, Yogev, Springer, Simon, & Giladi, 2005; Yogev‐Seligmann, Hausdorff, & Giladi, 2008). This theory agrees well with the result of a greater tendency in PD patients with dopamine‐unresponsive PIGD to develop dementia earlier in the disease course (Burn et al., 2006; Lewis et al., 2005). Notably, clarifying the association between executive deficits and PIGD may help us identify a common pathway of the neural networks jointly underlying cognitive processes and PIGD. In the current work, we have also found a correlation between TMT and bradykinesia/rigidity, which is in agreement with previous reports showing that the spatial pattern of fronto‐parietal hypometabolism was correlated with executive impairment tested by Trail Making Test Part B (Huang et al., 2007).

Attention comprises three different subdomains including executive control, orienting and alerting (Petersen & Posner, 2012), and is implicated in fronto‐parietal, corticopetal cholinergic and noradrenergic pathways. Overlapping this circuit are dopaminergic networks engaged with execution of tasks. In the current study, Symbol Digit Modality Test (SDMT) and TMT‐A as the measurements of attention were revealed to be associated with bradykinesia. An early positron emission tomography (PET) scan‐based study suggested that the performance in vigilance as an attention test was positively correlated with the Fluoro‐L‐DOPA uptake in the dorsolateral prefrontal cortex (Bruck, Aalto, Nurmi, Bergman, & Rinne, 2005), indicating the relationship between prefrontal cortex‐striatum dopaminergic systems and attention. In a previous report by our group, we showed that FDG‐PET imaging of PD patients with dementia (PDD) presented bilateral areas of hypometabolism in the frontal and posterior parietal‐occipital lobes compared with PD‐MCI patients and showed greater metabolic reductions in comparison with PD patients without dementia (Tang et al., 2016). This finding highlighted the role of frontal and parietal cortices in cognitive disturbance of patients diagnosed with PDD. Results of other reports also showed the association between attention and PIGD, in which fallers with PD presented worse performance of attention than non‐fallers (Allcock et al., 2009; Yogev et al., 2005). However, this correlation was not shown in the current study, suggesting or highlighting the complicated and heterogeneous interrelation of motor and cognitive symptoms in PD across different studies.

In the remaining subdomains of cognitive dysfunction in PD, copy time of CFT in visuospatial domain was shown to be associated with bradykinesia and recall score of CFT in the memory domain was associated with tremor and bradykinesia. To our knowledge, the latter correlation between visuospatial memory reservation and tremor has yet to be reported, and may reflect the involvement of other non‐dopaminergic transmitter systems, for example, the cholinergic system, well known for its role in memory (Gratwicke et al., 2015; Helmich, Hallett, Deuschl, Toni, & Bloem, 2012). Tremor in PD is believed to result from the disruption of two circuits: a dopaminergic striatopallidal circuit and a cerebello‐thalamo‐cortical circuit (Helmich et al., 2012), and may also partially originate from serotonergic dysfunction (Doder, Rabiner, Turjanski, Lees, & Brooks, 2003).

The discrepancies between different reports describing the relationships between cognitive dysfunction and motor impairments in PD may be explained by the following confounding factors: demographics, disease duration, medications, and cultural differences that may include the use of Chinese characters in language writing that might lead to better graphic information perception.

There are several limitations in this study or considerations to be made when interpreting the results. Firstly, PD patients recruited in the study were not drug naive, their motor functions were rated during off‐medication and cognition assessed during on‐medication. To this regard, there was concern that anti‐PD medications may affect neuropsychological evaluation as dopamine replacement therapy has previously been shown to produce both beneficial and aggravating effects on different cognitive functions (Cools, 2006). However, there were also caveats in newly diagnosed and drug naive PD patients in this type of study, since the motor and cognitive deficits may be mild and its narrow range might be difficult to be interrelated to other variables. Previous studies have been reported whereby these associations in medicated PD patients at various clinical stages was evaluated (Domellof, Forsgren, & Elgh, 2013; Williams et al., 2007). Secondly, neuropsychological tests, such as the TMT A/B, applied in this study may be directly influenced by Parkinsonian motor symptoms, also, the performance of verbal fluency tasks was affected by dysarthria in those advanced PD patients. We performed neuropsychological test during on‐medication state to minimize influence of motor dysfunction, however, the motor‐cognition correlations found between on‐off medication state might be spurious. Third, there were inevitably rater's bias and motor fluctuation in the subjective assessments of motor dysfunction. Finally, caution should be kept in mind to interpret the statistically significant findings. There could be many different causes for the correlation. For instance, the anti‐parkinsonism medication effect. Our assumption that two correlated functions are governed by shared underlying mechanism should be verified by further studies employing, for example, functional neuroimaging.

Future efforts should focus on longitudinal studies tracking the change of associations between the motor and cognitive variables over time. A similar one‐year follow‐up study was conducted that focused on bradykinesia and working memory, etc. in a clinical research setting. They showed that change in cognitive and motor functions were associated from time of diagnosis until 1 year after diagnosis (Domellof et al., 2013). Nevertheless, longer period of monitoring is warranted to better understand the neuropathophysiological origins of diverse motor and nonmotor signs in PD patients. Such outcomes could lead to better understanding of prognosis and bears the potential to facilitate discovery of new avenues for symptomatic treatments.

## Conflict of Interest

None of the authors report any financial interest or benefit arising from the direct application of this research. The authors have no conflict of interest to report.
